# A scoping review of practice recommendations for clinicians’ communication of uncertainty

**DOI:** 10.1111/hex.13255

**Published:** 2021-06-08

**Authors:** Niki M. Medendorp, Anne M. Stiggelbout, Cora M. Aalfs, Paul K. J. Han, Ellen M. A. Smets, Marij A. Hillen

**Affiliations:** ^1^ Department of Medical Psychology Amsterdam UMC Amsterdam Public Health Amsterdam The Netherlands; ^2^ Medical Decision Making Department of Biomedical Data Sciences Leiden University Medical Center Leiden The Netherlands; ^3^ Division of Biomedical Genetics Department of Genetics University Medical Center Utrecht Utrecht University Utrecht The Netherlands; ^4^ Center for Outcomes Research and Evaluation Maine Medical Center Research Institute Portland ME USA

**Keywords:** clinicians, communication, disclosure, health care, health personnel, physician‐patient relations, referral and consultation, review, uncertainty

## Abstract

**Background:**

Health‐care providers increasingly have to discuss uncertainty with patients. Awareness of uncertainty can affect patients variably, depending on *how* it is communicated. To date, no overview existed for health‐care professionals on how to discuss uncertainty.

**Objective:**

To generate an overview of available recommendations on how to communicate uncertainty with patients during clinical encounters.

**Search strategy:**

A scoping review was conducted. Four databases were searched following the PRISMA‐ScR statement. Independent screening by two researchers was performed of titles and abstracts, and subsequently full texts.

**Inclusion criteria:**

Any (non‐)empirical papers were included describing recommendations for any health‐care provider on how to orally communicate uncertainty to patients.

**Data extraction:**

Data on provided recommendations and their characteristics (eg, target group and strength of evidence base) were extracted. Recommendations were narratively synthesized into a comprehensible overview for clinical practice.

**Results:**

Forty‐seven publications were included. Recommendations were based on empirical findings in 23 publications. After narrative synthesis, 13 recommendations emerged pertaining to three overarching goals: (a) preparing for the discussion of uncertainty, (b) informing patients about uncertainty and (c) helping patients deal with uncertainty.

**Discussion and conclusions:**

A variety of recommendations on how to orally communicate uncertainty are available, but most lack an evidence base. More substantial research is needed to assess the effects of the suggested communicative approaches. Until then, health‐care providers may use our overview of communication strategies as a toolbox to optimize communication about uncertainty with patients.

**Patient or public contribution:**

Results were presented to stakeholders (physicians) to check and improve their practical applicability.

## 1 INTRODUCTION

Uncertainty is pervasive in medicine. It has been defined as a meta‐cognition—a self‐reflective mental state in which one is subjectively aware of one's ignorance.^1^ Rapid technological developments have yielded not only vast amounts of new biomedical knowledge but also information that may be difficult to interpret and/or overly complex.^2^ Moreover, the rise of evidence‐based medicine has paradoxically increased collective awareness within medicine of what is still unknown.^3^, ^4^, ^5^ For example, the meaning and implications of genetic mutations cannot always be known, it is unpredictable which treatment will best benefit individual patients, and diagnostic test results may be difficult to interpret.^5^, ^6^ Health‐care providers, patients and health researchers increasingly have to deal with these uncertainties.^7^

In parallel with growing awareness of uncertainty, patients’ roles have shifted: their information rights are being increasingly formally acknowledged. Moreover, there has been a rise in shared decision making, whereby health‐care providers are expected to involve patients in decisions about their health and treatment.^8^, ^9^ However, to properly inform patients and justify their autonomy, clinicians need to be fully open about what they do and do not know.^10^, ^11^, ^12^, ^13^, ^14^ In practice, this means they have to share with patients different types of uncertainty.

Both theory and research on uncertainty have been expanding in the past decades. The many manifestations of uncertainty have been approached from various disciplines, ranging from economics and mathematics to philosophy, psychology and sociology, which has resulted in wide variability in conceptual models and terminology.^1^, ^5^ Within health care, two main types of uncertainty are generally distinguished, albeit using various labels. First, first‐order uncertainty, probability or aleatory uncertainty refers to the inability to predict future outcomes regarding, for example prognosis, or treatment effects, and often involves using risk estimates.^1^ Second‐order or epistemic uncertainty can arise either from ambiguity or from complexity of information, and may for example concern the inability to interpret test results or to provide a definitive diagnosis.^15^ In 2011, Han^1^ proposed a comprehensive taxonomy of uncertainty in health care, distinguishing not only various *types*—or causes—of uncertainty, but also additionally different *issues* to which uncertainty may pertain (eg, scientific issues regarding diagnosis, prognosis, cause and treatment of a given medical condition, as well as practical and personal issues), and the *locus*—or person(s)—in whom the uncertainty resides.

Most empirical research on communicating uncertainty to patients has focused on effects and implications of risk communication (first‐order uncertainty).^5^ It has yielded specific recommendations on how to convey risk information, particularly in written form.^5^, ^16^, ^17^, ^18^ Yet, despite the significance of written information (eg, writing or drawing, information leaflets and websites), clinicians’ oral information provision during medical encounters is considered by patients as their most important information source.^19^, ^20^, ^21^ Research on interpersonal oral communication, specifically regarding second‐order uncertainty, is more scarce, and its results have been inconclusive.^22^ For example, studies have reported contradicting effects of physicians’ uncertainty expressions on patient satisfaction.^23^, ^24^, ^25^, ^26^, ^27^

These conflicting results may be explained by variation across health‐care providers’ communicative approaches to conveying uncertainty. Physicians’ expressions of uncertainty were found to be detrimental to patient satisfaction particularly if physicians did not perform actions to support patients in managing the uncertainty.^25^ In another study, clinicians’ *explicit* acknowledgement of uncertainty (eg, “I don't know”) was detrimental to patient confidence, whereas behaviours *implying* uncertainty (consulting a book or colleague) were seen as benign or even beneficial to trust.^28^ Apparently, discussing uncertainty with patients can have variable effects, depending on health‐care providers’ specific communication approaches, and providers may need to tailor their communication strategies according to the specific uncertainties at hand.

Despite general agreement that providers should discuss uncertainty with patients, to our knowledge no comprehensive guidelines are available for how to do so. This is problematic, as it could result in unwarranted practice variation in provider‐patient communication. As a result, patients might be exposed to suboptimal communication, inducing possible underrecognition, excessive awareness and/or misunderstanding of uncertainty.^16^ Eventually, this could result in feelings of uncertainty and anxiety, misunderstanding, impaired decision making and/or reduced satisfaction with and trust in their physician.^29^ We sought to create an initial overview of the practical recommendations on how health‐care providers can orally communicate uncertainty to patients within clinician‐patient encounters. We focused on second‐order uncertainty rather than publications focusing exclusively on first‐order uncertainty. Using a scoping review of the empirical and non‐empirical literature, we identified which practical recommendations are available for health‐care providers to discuss uncertainty with patients, and how evidence‐based these are.

## 2 METHODS

A scoping review was deemed the most appropriate type of review to meet our aim, as it enables exploring the breadth of existing research, comprehensively mapping the literature and providing directions for future research.^30^, ^31^ We developed the review protocol using the Preferred Reporting Items for Systematic reviews and Meta‐Analyses extension for Scoping Reviews (PRISMA‐ScR).^32^

### 2.1 Search strategy

A search strategy was set up in MEDLINE and then translated to CINAHL, EMBASE and PsycINFO (see Table 1). It included any variations of the following keywords: (1) clinical practice guideline, (2) communication, (3) uncertainty and (​4) health‐care providers. Note that “risk” and “risk communication” were deliberately not included in our search. We aimed to exclude the body of literature exclusively focused on risk as it often focuses on the more technical aspects of communicating statistical/numerical information (eg, using percentages vs frequencies). We searched databases from inception until 24 July 2019.

**TABLE 1:** Search strategy used in MEDLINE

1	Clinical practice guideline [MeSH]
2	(recommend* or advice* or advis* or tips or suggestion* or strategy or strategies or approach* or practice or principle* or skills or training or problems or (clinical adj1 practice adj1 (variation or pattern))).AB,TI,KF
3	OR/1‐2
4	Communication [MeSH]
5	(disclos* or communicat* or discuss* or conversat* or interact* or explain* or explanat* or ((provision or provid* or disseminat* or convey* or deliver* or exchang*) adj3 (information or result* or outcome or message*))).AB,TI,KF
6	OR/4‐5
7	Uncertainty [MeSH]
8	(uncertain* or doubt or ambigu*).AB,KF,TI
9	OR/7‐8
10	Healthcare professional/Physician [MeSH]
11	(((health care) adj1 (provider* or professional*)) or ((medical or health) adj1 (professional* or provider* or practitioner* or specialist)) OR ((primary care) adj1 (professional* OR physician* OR clinician*)) OR (family physician*) or doctor* or clinician* or health or medicine).AB,KF,TI
12	OR/10‐11
13	AND/3,6,9,12

### 2.2 Article selection and exclusion criteria

**FIGURE 1:**
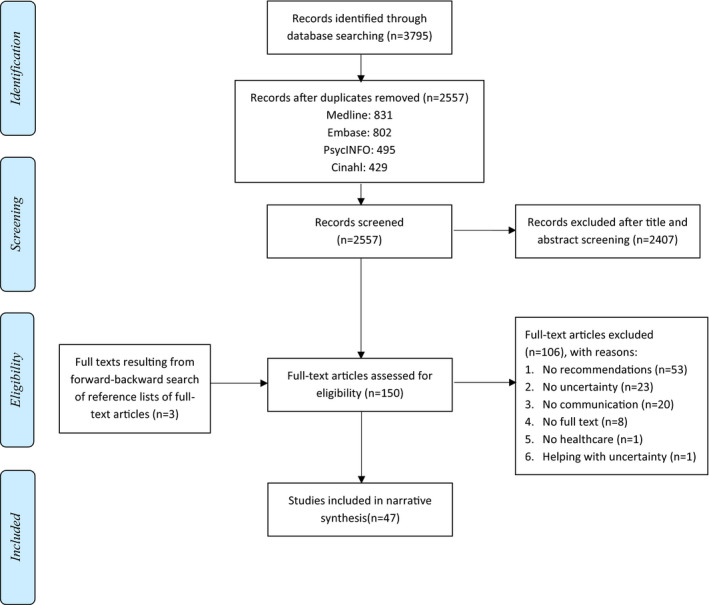
PRISMA flow diagram visualizing the article selection process

Figure 1 illustrates the article selection process. Three reviewers (EvB, NM and MH) screened all titles and abstracts for eligibility, using Rayyan,^33^ in three steps: first, 50 abstracts were screened jointly to further specify inclusion and exclusion rules; second, all three reviewers independently screened 200 abstracts and solved any discrepancies; and third, the remaining abstracts were independently screened by two reviewers each. Discrepancies were solved through discussion. Any types of English/Dutch‐written abstracts (including dissertation abstracts, non‐empirical papers) were included if describing any type of recommendation—evidence‐based or not—for any type of health‐care provider on *how to* (not *whether to*) orally communicate uncertainty to patients. Articles were excluded if they (1) included only an abstract (eg, conference proceedings); (2) included only recommendations for providers to *help patients deal with* uncertainty; and (3) concerned only risk communication in its narrowest sense.

Full‐text screening was performed by two (out of three) reviewers independently, using the same criteria. Any discrepancies were discussed and resolved during consensus meetings. Reference lists of all papers included after full‐text screening were checked for additional relevant papers.

### 2.3 Data extraction

Two reviewers (NM and MH) performed data extraction using an instrument based on the PRISMA and RAMESES guidelines.^34^, ^35^ Data from the first three articles were extracted jointly to fine‐tune the extraction form. The remaining data were extracted by one reviewer each, and any doubts were discussed. Aside from descriptive characteristics for each study (eg, year of publication, publication type, design and population), we extracted any recommendations regarding the communication of uncertainty that were provided. For these recommendations, we specified (1) medical setting and context/topic to which the recommendations applied (eg, diagnosis, treatment decision); (2) target group, that is for which health‐care providers the recommendations were intended; and (3) strength of the evidence base (ie, whether findings were based on new or previous empirical findings, non‐empirical literature or no evidence). Assessment of these criteria was inductive, meaning that no pre‐specified criteria were used, but rather these were allowed to emerge from the data.^36^ No quality assessment of the empirical studies was performed, as the aim was to provide a scoping overview rather than a systematic appraisal.

### 2.4 Narrative synthesis

After completing data extraction, the two reviewers reviewed and rephrased and/or summarized the recommendations, to enable comparison and aggregation of overlapping recommendations. A first draft of a list with recommendations was created and modified after review and discussion between authors (NM, AS, ES and MH). Moreover, the list was discussed with two additional authors—one a general internist/palliative care physician (PH) and the other a clinical geneticist (CA)—to check and improve their practical applicability. Finally, the list was finalized (see Table 3).

## 3 RESULTS

### 3.1 Publication selection

Figure 1 displays the publication selection process.^32^ The search yielded 2257 non‐duplicate references. Of these, 150 remained after screening of titles and abstracts, 106 of which were excluded based on full‐text screening. Three publications were added after backwards searching. In total, 47 publications were included in our narrative synthesis.

### 3.2 Characteristics of publications

Table 2 provides an overview of the publications key characteristics and a summary of the provided recommendations. All except two older publications were published between 2002 and 2019, and they originated from eleven different countries: USA (n = 23), UK (n = 7), the Netherlands (n = 5), Australia and Canada (both n = 3), Belgium, France, Italy, Japan, Norway and New Zealand (all n = 1). Of 26 publications describing empirical work, 14 were qualitative studies, involving interviews (n = 8) or observations of consultations (n = 6). Quantitative study designs involved experimental (vignette) studies (n = 5), cross‐sectional studies (n = 3), quantitative observational studies (n = 2), one intervention and one mixed‐methods study. Non‐empirical publications included conceptual papers (n = 5), opinion papers (n = 5), reviews (n = 5), practical recommendations (n = 4), one review/case study and one comment. If the medical setting was specified (32/47 publications), it most commonly involved oncology (n = 8), clinical genetics (n = 7) and paediatrics (n = 3). In 25 articles, recommendations were targeted at “physicians” in general, whereas other publications addressed particular disciplines or health‐care providers.

**TABLE 2:** Key characteristics of included articles in alphabetical order

First author, year (country)	Article type *Study design/subtype*	Medical setting	Context of recommendation	Target group	Recommendations supported or implied by empirical evidence^a^	Recommendation(s)
Ahalt, 2012 (USA)^46^	Empirical *Qualitative interview study with older patients who receive help to live at home*	n/p^b^	Prognosis	Physicians	Empirically implied: based on older patients’ preferences for discussing prognosis	Explore patients' preferences for receiving prognostic information Discuss prognostic information in a direct and empathic manner, and be willing to spend time on its discussion Acknowledge that any prognosis is uncertain
Alby, 2017 (Italy)^63^	Empirical *Qualitative observational study of communication strategies in oncological consultations*	Oncology	Decision making about treatment options	Oncologists	Empirically supported: specific communication strategies were observed in clinical practice	Alternate uncertain bad news with certain good news, to create a balance between accurate information giving and the need for reassurance Limit the discussion of future treatment choices to a discrete set of logically constrained options. Take the patient along in your reasoning to enhance their knowledge and insight into the decision making process Use your personal professional wisdom to make predictions about test outcomes to enhance reassurance
Armstrong, 2018 (USA)^56^	Non‐empirical *Opinion*	n/p^b^	Decision making	Physicians		Discuss uncertainty in an empathetic, positive manner that partners the physician‐patient relationship Avoid using single numbers as this may lead to misleading about levels of precision. Use numerical ranges with qualitative estimates instead Regarding diagnostics, emphasize that test results shift probabilities up or down rather than that they provide a definitive answer Emphasize your continued involvement in clinical care
Berger, 2015 (USA)^55^	Non‐empirical *Opinion*	n/p^b^	Shared decision making about treatment options	Physicians		Be honest and acknowledge the limits to your knowledge or the guidelines Acknowledge and identify emotions and other, non‐logical modalities when discussing uncertainty in decision making, to enhance patients' satisfaction Provide hope by envisaging a positive outlook in the face of uncertainty, to help patients navigate uncertainty Present all available options regarding patients' coordination of care and further support, when course and outcomes of illness are uncertain, and involve the patient in decisions regarding coordination of care Show willingness to readdress certain topics, goals or options at a later stage if the patients' emotions about it change, or if new information is available that reduces uncertainty or changes options
Bhise, 2018 (USA)^61^	Empirical *Experimental video vignette study among parents of paediatric patients comparing communication strategies*	Paediatrics	Diagnosis	Physicians	Empirically supported: effects of specific communication strategies (explicit vs implicit communication of uncertainty) on patients’ trust and perception of technical competence were compared	Be implicit in conveying the uncertainty associated with your diagnosis, for example by using the most likely or differential diagnosis instead of explicitly acknowledging that you are “not sure”
Blanch‐Hartigan, 2019 (The Netherlands)^27^	Empirical *Experimental video vignette study among (former) cancer patients comparing communication strategies*	Oncology	Diagnosis^c^	Physicians	Empirically supported: effects of specific communication strategies (high vs low expression of non‐verbal uncertainty) on patients’ trust were compared.	Be careful not too visibly express uncertainty non‐verbally, such as by stammering
Brookes‐Howell, 2006 (UK)^58^	Empirical *Qualitative observational study of communication strategies in genetic consultations*	Clinical genetics	Diagnosis	Genetic counsellors	Empirically supported: observation of specific communication strategies and their apparent effect on patient/parent behaviour	Be explicit about the level of evidence (or the lack thereof) about diagnosis Express hope of eventually finding a diagnosis Discuss the potential impact of possible diagnostic scenarios with the patient Provide a provisional diagnosis to patients to explain their disease to other people Acknowledge the patients' need for certainty
Cagle, 2016 (USA)^59^	Empirical *Qualitative interview study with caregivers of disabled older adults*	Geriatrics	Prognosis	Physicians working with elderly	Empirically implied: based on caregivers’ preferences for being included in prognostic conversations	Be honest about the degree of accuracy of a prognostic estimate Provide hope by emphasizing the controllable, for example patients maintaining independence by using assistive devices Convey your expectations as best as possible regarding disease trajectory, care needs and ways to preserve independence
Carter, 2018 (UK)^48^	Empirical *Qualitative interview study with women at risk of pre‐term labour*	Midwifery	n/p^b^	Physicians working with women at risk of pre‐term labour	Empirically supported: women's reports of how they were affected by specific communication strategies	Try to minimize conflicting advice Assess people's individual coping strategies and adapt the level of detail in your information provision accordingly
Engelhardt, 2014 (The Netherlands)^49^	Non‐empirical *Review*	Oncology (breast cancer)	Prognosis	Oncologists		Explore patients' individual preferences regarding prognostic information, and adapt your level of precision accordingly
Fisher, 2012 (Australia)^80^	Non‐empirical *Opinion*	End‐of‐life care	Prognosis	Physicians working in end‐of‐life care		Provide “practical uncertainty” by recognizing scientific uncertainty and reassuring the family that you are as certain as you can be
Gheihman, 2019 (USA)^73^	Non‐empirical *Practical recommendations*	Medical education in general	Uncertainty in general	Physicians		Make clear to the patient that you will provide support through the process even when the clinical answer is uncertain
Griffiths, 2005 (UK)^81^	Empirical *Qualitative observational study of communication strategies in consultations in primary and secondary care*	n/p^b^	Decision making about treatment options	Physicians	Empirically supported: observation of specific communication strategies in practice	Try to come to provisional decisions together with the patient to create some form of certainty
Han, 2013 (USA)^16^	Non‐empirical *Conceptual paper*	Health care in general	Clinical evidence	Physicians		Tailor the communication of uncertainty according to patient preferences for information and their tolerance of uncertainty Provide support by responding to emotions resulting from the uncertainty
Han, 2019 (USA)^5^	Non‐empirical *Conceptual paper*	Health care in general	Uncertainty	Physicians		Tailor the degree of uncertainty and associated detail you communicate to your type of goal: for example, if it is to provide more autonomy, you should communicate more uncertainty with higher precision than if you aim to persuade the patient
Holloway, 2013 (USA)^72^	Non‐empirical *Conceptual paper*	Neurology	Prognosis	Neurologists^c^		Show commitment by conveying your availability and continued support to the patient
Johnson, 1988 (USA)^25^	Empirical *Experimental video vignette study among patients comparing communication strategies*	Ambulatory care	Decision making about treatment options	Physicians	Empirically supported: effects of specific communication strategies (ignoring/acknowledging uncertainty; acting upon uncertainty) on patients’ satisfaction were compared	Convey uncertainty using calm, reassuring and empathic communication—appearing untroubled, and in control Appear concerned for the patient, make clear you have their optimal care as your highest priority
Johnson, 2013 (USA)^50^	Non‐empirical *Review/case study*	Acute care (ICU)	Prognosis and uncertainty in general	Physicians working in ICU and in general		Assess people's coping strategy, particularly whether they see uncertainty as a source of hope or as a threat Contrast the hope and worry aspects of the situation Tailor the amount of uncertainty communication to patients' and their loved ones' individual perceived needs
KirkebØen, 2019 (Norway)^57^	Empirical *Two written vignette‐based studies: (1) an observational study of communication strategies; and (2) an experimental study among students comparing communication strategies*	Oncology	Prognosis	Physicians	Empirically supported: effects of specific communication strategies (framing of prognosis) on patients’ optimism and realistic understanding of prognosis were compared	Be realistic by explaining that you can provide information for a group rather than an individual Provide hope by communicating uncertain prognosis in a positive, hopeful manner (“Half of the patients will live longer than the mean”) and give concrete example of these survivors
Lemmon, 2016 (USA)^65^	Empirical *Qualitative interview study with parents*	Neonatology	Prognosis	Physicians working in NICU	Empirically implied: based on exploration of parents’ experiences regarding communication with physicians	Avoid overly vague prognostication, and provide best, worst and most likely outcome scenarios
Leydon, 2008 (UK)^82^	Empirical *Qualitative observational study of communication strategies in oncological consultations*	Oncology	Treatment options	Physicians	Empirically supported: observation of specific communication strategies in clinical practice	Use “proximal pairing”, that is following uncertain or bad news with good news, because it may reduce the burden of uncertainty. But use it with caution because it might reduce space for patient concerns
Libert, 2016 (Belgium)^71^	Empirical *Quantitative study of observations of communication strategies and survey data*	Health care in general	Decision making	Oncologists	Empirically implied: based on assessment of physician satisfaction about their management of uncertainty in simulated consultation	When communicating uncertainty, facilitate patients' emotional expression and provide them with emotional support
MacEntee, 2014 (Canada)^44^	Non‐empirical *Review*	Deontology	Informed consent (about treatment)	Gerodontologists^c^		When asking informed consent, prepare patients for disappointment and provide the opportunity to adapt to and cope with the unexpected. Do so by using stories of concrete, emotionally interesting information (ie, narrative) rather than through factual assertions of abstract data or statistical evidence; and by segmenting problems, identifying choices and clarifying probabilities
Maguire, 1988 (UK)^70^	Non‐empirical *Practical recommendations*	Oncology	Prognosis	Oncologists^c^		When conveying prognostic uncertainty, provide patients with a sense of control by giving guidelines about what to do and what to expect in the coming time Explore any concerns that the patient has as a result of the uncertainty
Makhnoon, 2018 (USA)^38^	Empirical *Three studies: 1) an interview study with cancer patients; and 2) two quantitative survey studies among counselees*	Clinical genetics	Genetic testing	Genetic counsellors	Empirically supported: patient reports of their experiences with physicians’ specific communication strategies	Prepare patients for the possibility of uncertain outcomes
Medendorp, 2018 (The Netherlands)^68^	Empirical *Qualitative observational study of communication strategies in genetic consultations*	Clinical genetics (cancer)	Genetic testing	Genetic counsellors	Empirically supported: observation of specific communication strategies in clinical practice	When discussing uncertainty, summarize key points, check patients' understanding and take patients' personal uncertainties into account
Medendorp, 2019 (The Netherlands)^37^	Empirical *Qualitative interview study with counsellors and counselees*	Clinical genetics (cancer)	Multigene panel testing	Genetic counsellors	Empirically implied: based on counsellors and counselees’ in‐depth accounts of their experiences with uncertainty	Discuss potential uncertain outcomes When discussing uncertainty, slowly pace the information
Mori, 2019 (Japan)^51^	Empirical *Quantitative cross‐sectional survey study among cancer patients comparing communication strategies*	Oncology	Prognosis	Physicians	Empirically supported: effects of specific communication strategies (framing of prognosis) on patients’ preferences were compared	When discussing prognostic information, disclose explicit information, provide both best and worst scenarios and a range in survival time Assess patients' coping style and information preferences to tailor prognostic information or avoid explicit prognostic information (when patients' coping strategies require so) Explain why accurate prognostication is difficult and discuss what can be done in the face of uncertainty
Newson, 2016 (Australia)^43^	Non‐empirical *Conceptual paper*	Genomics	Genomic testing	Physicians working in genomics		Prepare the patient for uncertainty by avoiding the use of the rhetoric “test to reduce uncertainty” as reducing uncertainty may not necessarily be the case When discussing uncertainty, explain the source and reliability of uncertain information and offer appropriate follow‐up Identify, together with patients, suitable coping strategies and responses
Ogden, 2002 (UK)^28^	Empirical *Quantitative cross‐sectional survey study among general practitioners and patients comparing communication strategies*	n/p^b^	Diagnosis and management aspects	General practitioners	Empirically supported: physicians’ and patients’ hypothetical reports on how specific communication strategies would affect them	Avoid use of explicit statements of uncertainty such as “I don't know” Use behavioural expressions of uncertainty such as asking the advice of other doctors
Olsen, 2018 (USA)^69^	Empirical *Evaluation of an intervention targeted at residents’ communication skills*	Paediatrics	Diagnosis	Residents^c^	Empirically supported: learning points made by physicians participating in a training programme for responding to clinical uncertainty	When discussing uncertainty, describe the diagnostic process in appropriate but not excessive detail, use empathy, avoid false reassurance and ensure a clear plan for follow‐up
Parascandola, 2002 (USA)^14^	Non‐empirical *Opinion*	n/p^b^	Decision making	Physicians		Discuss the reason for the uncertainty so as to avoid confusion, with a level of ease and comfort Encourage patients to seek support
Pighin, 2011 (France)^67^	Empirical *Two quantitative experimental studies, among students and pregnant women comparing communication strategies*	n/p^b^	Providing bad news^c^	Physicians^c^	Empirically supported: effects of specific communication strategies (using vs not using qualifiers when conveying good vs bad news) on patients’ confidence in information	When expressing uncertainty, particularly regarding bad news, avoid any form of vagueness, which could be read by patients as either hedging or indirectness, and routinely check patients' understanding
Pino, 2019 (UK)^52^	Empirical *Qualitative observational study of communication strategies in hospice consultations*	n/p^b^	Prognosis	Physicians	Empirically supported: observation of patients’ and physicians’ communication strategies in clinical practice	Ask about the patient's knowledge on their life expectancy, invite their perspective on (eg, how they feel about) life expectancy and check their readiness to know more about their life expectancy
Polaner, 2017 (USA)^60^	Non‐empirical *Comment*	Paediatrics	Decision making	Physicians		Be cautious about advising about the risk of uncertain outcomes, when clinical data is uncertain
Portnoy, 2013 (USA)^45^	Empirical *Quantitative cross‐sectional survey study among physicians*	n/p^b^	Uncertainty and decision making^c^	Physicians	Empirically implied: physicians’ self‐report of their own and patients’ management of uncertainty	Talk about how to address uncertainty instead of letting your (possibly inaccurate) perceptions of patients' ambiguity aversion influence your communication of uncertainty or, for example, your reluctance towards such communication
Ridley, 2013 (Australia)^83^	Non‐empirical *Review*	End‐of‐life care	Prognosis	Physicians^c^		Present prognosis in terms of “Practical certainty”—explaining that according to the opinions of a number of people you can be as certain as you can be about it
Roberts, 2016 (USA)^39^	Empirical *Qualitative interview study with oncologists*	Oncology	Oncotype DX testing	Physicians^c^	Empirically implied: physicians’ in‐depth self‐report about their own communication strategies regarding genetic testing	Prepare patients for the possibility of uncertain outcomes Develop management plans to prepare patients for uncertainty in case of specific outcomes
Santhosh, 2019 (USA)^64^	Non‐empirical *Opinion*	n/p^b^	Diagnosis	Trainees^c^		Even when acknowledging uncertainty, be as specific as possible by listing potential diagnoses Check the emotional impact of the uncertainty on the patient, and respond to it using empathy and shared goals Outline a clear follow‐up plan: make sure what the next steps are to try to reduce uncertainty
Schwab, 2012 (USA)^66^	Non‐empirical *Conceptual paper*	n/p^b^	Decision making (informed consent)	Physicians		When explaining the evidence base for a recommendation, be explicit about how certain it is, using plain terms (and possibly even an analogy to facilitate understanding)
Seely, 2013 (Canada)^53^	Non‐empirical *Conceptual paper*	Health care in general	Diagnosis, prognosis and treatment^c^	Physicians		Explain the next steps (diagnostic tests) that will be taken to reduce uncertainty Highlight which uncertainties (diagnostic, prognostic, therapeutic) are irreducible Assess patients' beliefs and values in order to adapt your communication of uncertainty to the individual
Simpkin, 2019 (USA)^47^	Non‐empirical *Review*	n/p^b^	Decision making	Physicians		Explicitly assess patients’ desire for information and method of delivery for that information, and tailor the communication accordingly Provide emotional support and emphasize your continued involvement in care Explain the degree and nature of uncertainty, and explain its complexities Provide a clear follow‐up plan
Skinner, 2018 (USA)^42^	Empirical *Ethnographic study including observation of consultations and qualitative interviews with patients and their parents*	Genomics	Genomic testing	Genetic counsellors	Empirically supported: observation of specific communication strategies and their effects on patient understanding and evaluation	Prepare patients for the possibility of an uncertain or uninformative outcome of a genetic test—thus managing their expectations Emphasize that uncertain results are neither positive nor negative Make clear that informing patients about uncertain test results enables possible future re‐interpretation
Solomon, 2017 (USA)^40^	Empirical *Qualitative interview study with individuals who received a variant of unknown significance*	Clinical genetics	Variants of unknown significance	Physicians^c^	Empirically implied: counselees’ experiences with receiving an uncertain test result	Prepare patients for the possibility of an uncertain test outcome Anticipate on and respond to patients' emotional reactions to uncertainty Provide structure to help patients make meaning of uncertain results Help prepare patients for uncertainty: provide clear plans for follow‐up care and management recommendations Make a clear plan with patients for follow‐up regarding reclassification updates
Stortenbeker, 2019 (The Netherlands)^62^	Empirical *Quantitative observational study in general practitioner consultations about MUS*	Family medicine	Medically unexplained symptoms (MUS)	General practitioners	Empirically supported: cross‐sectional analysis examining relation between specific strategy (implicit communication of uncertainty) and patient anxiety	Don't hesitate to use implicit or subtle ways to demonstrate uncertainty (eg, “maybe”, “it could be”)—because these merely reflect the complexity of communicating a diagnosis
Than, 2009 (New Zealand)^41^	Non‐empirical *Practical recommendations*	n/p^b^	Diagnosis	Physicians		Prepare patients for the possibility of an uncertain outcome
Zizzo, 2017 (Canada)^54^	Non‐empirical *Practical recommendations*	Family medicine	Uncertainties related to foetal alcohol spectrum disorder (FASD)	General practitioners		Tailor your message to individuals appropriately

^a^ Column indicates for all empirical studies whether the recommendations were directly derived from the study findings (eg, when different communication strategies were observed or compared), or indirectly based on study findings (eg, when clinical geneticists’ views on uncertainty were explored).

^b^ n/p means that the information was not provided by the publication.

^c^ Information was not specified in the article but derived from other, additional information mentioned in the article.

### 3.3 Characteristics of recommendations

Recommendations for communicating uncertainty were explicitly stated to apply to the following context: communicating prognosis (n = 12), (shared) decision making (n = 12), conveying diagnosis (n = 6) or genetic/genomic testing (n = 6). Seven recommendations applied to discussing uncertainty in general. In four publications, the context of the recommendation(s) was not explicitly stated but inferred by the authors from other information in the article (eg, setting). In 23 publications, evidence directly obtained from empirical work reported in the paper led to the provided recommendations. Thirteen of these could be considered “empirically supported”, that is directly supported by study findings, for example when different strategies for conveying uncertainty were observed or compared. Ten were “empirically implied”, that is more indirectly deduced from the study results, for example when communication preferences were identified in interviews with clinicians. In the remaining 24 publications, recommendations were based on previous (non‐)empirical literature (n = 19), or not substantiated by any literature (n = 5).

### 3.4 Narrative synthesis of recommendations

From our narrative synthesis, 13 recommendations emerged addressing three overarching goals: (a) preparing for the discussion of uncertainty, (b) informing patients about uncertainty and (c) helping patients deal with uncertainty (see Table 3). Below, we discuss all recommendations, and which data acquired in empirical research reported in the publication itself supports them.

**TABLE 3:** A concise overview of all recommendations on communicating uncertainty

Overarching aim	Recommendation	Explanation and examples
Preparing for the discussion of uncertainty	1. Warn patients for the possibility of uncertain outcomes	Before initiating treatment or diagnostic/genetic testing, prepare patients that they may be confronted with uncertain outcomes.^37^, ^38^, ^39^, ^40^, ^41^ This should help patients manage their expectations,^42^ and prepare them for continued uncertainty,^43^ disappointment^44^ and uninformative test outcomes^42^ “Many things are uncertain and unpredictable right now—this means I do not have all the answers for you”
	2. Explore patients’ individual preferences, beliefs and coping styles regarding uncertainty and adapt your communication accordingly	Actively explore how patients see uncertainty (eg, as a source of hope or as threat), how they deal with uncertain situations, and what their preferences are for knowing uncertain information (eg, prognostic estimates). Do not make any assumptions about people's uncertainty tolerance.^45^ Tailor the level of detail of your information about prognosis or any other uncertainty to these individual styles and preferences^5^, ^54^ “Do you want to hear more about your prognosis or preferably not right now?”
Informing patients about uncertainty	3. Openly acknowledge inherent uncertainty and explain the degree and nature of available evidence	Be open and honest about any limits to the available knowledge that cannot be eliminated at this moment,^55^, ^57^ or that are irreducible by definition.^47^, ^53^ This may entail explaining that according to the available knowledge you can be as certain as you can be about it. When discussing prognosis, be explicit about the (in)accuracy of your estimates^46^, ^58^ and explain why accurate prognostication is difficult.^51^ When discussing diagnosis and/or diagnostic testing, explain the degree and nature of evidence (or lack thereof)^43^, ^56^, ^59^, ^60^ “Unfortunately we cannot definitively say what causes your complaints. There is no test to provide absolute certainty about this”
	4. Allow flexibility in the extent to which uncertainty is communicated, depending on the individual and circumstances	Be aware that for some patients and depending on circumstances, awareness of uncertainty may lead to negative emotions (eg, anxiety, feelings of uncertainty) and/or a more negative appraisal of their care provider. This strategy requires taking into account patient preferences and psychological capacity to tolerate uncertainty and adjusting one's communication accordingly. For example, when sensing that patients react negatively to explicit statements of uncertainty such as “I don't know”, it may be better to resort to more implicit means that effectively reflect the complex reality. Examples are as follows: explaining the most likely diagnoses,^61^ asking other doctors for advice^28^ and using careful terms such as “maybe”^61^, ^62^ “It is possible that the test shows you have this disease”
	5. Outline all potential scenarios and discuss their implications for patients’ life	Based on your expertise and the available knowledge, make predictions or draw preliminary conclusions.^58^, ^59^, ^63^ Specifically, discuss a discrete set of potential diagnostic/prognostic scenarios or treatment options.^64^ For prognostic communication, this is best done by providing best case, worst case and most likely scenarios, which may facilitate a sense of hope.^51^, ^65^ Take the patient along in your reasoning to enhance insight and knowledge.^63^ Outline the potential impact of each scenario on the patient's life^58^ “We cannot predict how your condition will develop, but there are roughly three scenarios: scenario 1 …” “In the worst case scenario […], in the best scenario […]. But the most probable scenario is […]”
	6. Explain uncertainty in an understandable, concrete and structured way	Explain uncertainty using understandable language, possibly even using analogies to facilitate understanding.^66^ Use concrete and emotionally engaging narratives instead of factual and abstract assertions.^44^ Provide structure by summarizing key points and slowly pacing the information.^37^, ^40^, ^67^, ^68^ Minimize conflicting advice and any form of vagueness^48^, ^67^ “I will tell you only the most important things right now. If you need more details, please feel free to ask me”
	7. Use non‐verbal communication that conveys confidence	When verbally communicating uncertainty, use non‐verbal communication that conveys ease, calm, reassurance and comfort, such as a calm voice and bodily posture.^14^, ^25^ Avoid non‐verbal signs of uncertainty such as less fluent or hesitant speech^27^
	8. Check patients’ understanding of the uncertainty	Explicitly check patients’ understanding of the provided uncertainty^67^, ^68^ “I want to know if I explained it clearly. What are the most important things you took away from what I told you about the genetic test?”
Helping patients deal with uncertainty	9. Identify, together with patients, suitable coping strategies, management plans and responses to future uncertainty	Help patients deal with future uncertainty, for example resulting from diagnostic or genetic testing. Prepare management plans together (eg, clear plans for follow‐up care), identify suitable coping strategies (eg, emphasizing continuity of care regardless of uncertainty) and/or develop suitable responses to uncertainty (eg, which family members to inform of a genetic test result).^39^, ^40^, ^43^, ^44^ Show willingness to readdress certain topics if new information becomes available that reduces uncertainty^55^ “What would help you in dealing with this uncertain situation? For example, some people like to talk about it a lot with friends and family, while others prefer not thinking about it too much. What's that like for you?”
	10. Provide some sense of control	Particularly when conveying diagnosis or prognosis, provide a sense of control to patients. This can be done by emphasizing the controllable elements of the situation, or providing guidelines about what to do and what to expect.^51^, ^59^, ^69^, ^70^ Particularly in case of an uncertain diagnosis, ensure a clear plan forward to reduce uncertainty^47^, ^53^, ^64^, ^69^ “How your symptoms develop is something you can unfortunately not control. What you can do is […]” “This is what we will do to try to get more certainty about what is causing your symptoms”
	11. Provide hope	Provide hope to patients by alternating uncertain bad news with certain good news,^50^, ^63^, ^82^ envisaging a positive outlook (eg, hope of eventually finding a diagnosis) in the face of uncertainty,^55^, ^58^ emphasizing that uncertain results are neither positive nor negative,^42^ communicating uncertain prognosis in a positive, hopeful manner^57^ “I’m afraid we cannot predict the course of your disease. What we do know is that the treatment is working, which is a positive thing”
	12. Facilitate patients’ emotional responses to the uncertainty, and provide emotional support	Facilitate patients’ emotional expressions to the uncertainty, explicitly check the impact it has on patients, which may include (a) acknowledging the personal uncertainties patients may have and their need for certainty,^58^, ^68^ and/or (b) providing emotional support according to patients’ needs using empathy and shared goals^40^, ^46^, ^47^, ^56^, ^64^, ^69^, ^70^, ^71^ “Many people find it difficult to hear there is so much uncertainty. How do you feel about hearing all of this?”
	13. Emphasize continued involvement with the patient's care	Show commitment by appearing concerned with the patient and confirming your continued support and availability throughout the process^25^, ^47^, ^56^, ^72^, ^73^ “Even though we are limited in our possibilities to help your husband fight the disease, we will remain highly involved in his care”

#### 3.4.1 Preparing for the discussion of uncertainty

We identified two recommendations aimed at preparing for the discussion of uncertainty. Eight publications included the suggestion that (1) providers should warn patients for the possibility of uncertain outcomes.^37^, ^38^, ^39^, ^40^, ^41^, ^42^, ^43^, ^44^ This recommendation applied particularly to situations involving future uncertainty, that is involving diagnostic or genetic testing. Only two publications described empirical studies directly supporting this recommendation, both reporting that patients appreciated if they were prepared for potential uncertainty resulting from genetic testing.^38^, ^42^

The recommendation to (2) explore patients’ individual preferences, beliefs and coping styles regarding uncertainty and to adapt communication accordingly was (re)iterated in 11 publications.^5^, ^54^ In two cases, this recommendation was empirically supported. One observational study found that cancer patients with a more active problem‐solving coping style preferred receiving explicit prognostic information from their physician.^51^ In a second observational study, women at risk of pre‐term birth varied in their preferences for receiving additional information to reduce uncertainty.^48^

#### 3.4.2 Informing patients about uncertainty

We distinguished six recommendations aimed at conveying uncertainty to patients. Eleven publications included the advice to (3) openly acknowledge inherent uncertainty and explain the degree and nature of the evidence that is available.^43^, ^60^ This may be viewed as a “double‐barreled” recommendation: clinicians may or may not add an explanation about the level of evidence to their acknowledgement of uncertainty. This recommendation particularly applied to diagnostic or prognostic settings and was supported by empirical data from three studies. In the first, caretakers of disabled older patients reported to prefer if uncertainty was openly discussed with them.^59^ The second concluded, based on qualitative observations, that people receiving genetic counselling were able to actively participate in handling an uncertain diagnosis.^58^ The third, experimental, study found that cancer patients had a more realistic understanding of the variation in survival time if possible variation in prognostic expectancies was explicitly discussed.^57^

Three publications suggested that (4) providers should allow flexibility in the extent to which uncertainty is communicated, depending on the individual and circumstance. This strategy requires taking into account patient preferences and psychological capacity to tolerate uncertainty and adjusting one's communication accordingly, for example by using more implicit wording (eg, “it could be” rather than “I don't know”).^28^, ^61^, ^62^ All three publications provided empirical support for this recommendation. Both a survey and an experimental study found that physicians’ explicit (vs implicit) expressions of uncertainty reduced patient‐perceived technical competence, trust, confidence and patient adherence.^28^, ^61^ A third (observational) study reported that general practitioners’ *implicit* expressions of uncertainty did not affect patients’ anxiety.^62^

The advice to (5) outline all potential scenarios and discuss their implications for patients’ life was put forth in six publications.^51^, ^58^, ^59^, ^63^, ^64^, ^65^ This recommendation applied mainly to the diagnostic phase, that is when a definitive diagnosis cannot yet be made or when discussing the potential outcomes of diagnostic testing. Two observational studies provided empirical support. In one, oncologists were observed to discuss possible scenarios of diagnostic test outcomes, thus providing patients with insight into their reasoning.^63^ In the second study, cancer patients reported to prefer prognostic information to be framed in terms of a best case, worst case and most likely scenario, compared with framings not including a wide range.^51^

In total, seven publications included recommendations to (6) explain uncertainty in an understandable, concrete and structured way, for example by using narratives.^37^, ^40^, ^44^, ^48^, ^66^, ^67^, ^68^ This recommendation was not directly empirically supported. Three publications included the recommendation that (7) when communicating uncertainty, providers should do so using non‐verbal communication that conveys confidence, for example by using a calm voice and avoiding stammering.^14^, ^25^, ^27^ This advice was supported empirically by one experimental study indicating that patients’ trust in an oncologist was reduced by non‐verbal expressions of uncertainty.^27^ Finally, two publications suggested that (8) providers should check patients’ understanding of the uncertainty.^67^, ^68^ One indirectly substantiated this claim with experimental evidence showing that physicians’ use of qualifying terms that conveyed uncertainty would sometimes be misinterpreted by patients.^67^

#### 3.4.3 Helping patients deal with uncertainty

Five recommendations pertained to the elements of uncertainty communication that support patients’ emotions and the physician‐patient relationship. Five publications suggested to (9) help patients identify suitable coping strategies, management plans and responses to future uncertainty, particularly in situations involving diagnostic or genetic testing.^39^, ^40^, ^43^, ^44^, ^55^ Examples are to make a concrete plan for follow‐up care, or explicitly emphasize a willingness to readdress topics if new information becomes available. No empirical support was provided for this recommendation.

In seven publications, it was suggested that clinicians should (10) provide patients and their close ones with some sense of control to counterbalance the uncertainty, particularly when conveying diagnosis or prognosis.^47^, ^51^, ^53^, ^59^, ^64^, ^69^, ^70^ This may involve highlighting that while one cannot control circumstances, one can do one's best in any scenario to choose the optimal path forward. Evidence on *which* interventions might be most effective in providing control is lacking, but two observational studies proposed potential interventions. Two observational studies showed that both caretakers of disabled older patients and paediatric residents saw the benefits of emphasizing the controllable elements of an uncertain situation.^59^, ^69^

Seven publications suggested to (11) provide a sense of hope to patients and/or their close ones, for example, by alternating uncertain bad news with certain good news, particularly in serious illness.^50^, ^57^, ^58^, ^59^, ^63^, ^64^, ^65^ Two empirical studies provided direct support. In one, caretakers of disabled elders suggested that physicians should help them maintain a sense of hope to deal with uncertainty.^59^ In the second, oncologists were observed to communicate uncertainty by continually alternating between uncertain (possibly) bad news and good, reassuring news—although no evidence was provided to validate this approach.^63^

Ten publications included the suggestion to (12) facilitate patients’ emotional responses to the uncertainty, and/or provide emotional support.^40^, ^71^ This recommendation was not empirically supported. Lastly, the advice for providers to (13) emphasize their continued involvement with the patient's care was (re)iterated in five publications, although it was not empirically supported.^25^, ^47^, ^56^, ^72^, ^73^

## 4 DISCUSSION

We reviewed the literature to identify practical recommendations for health‐care providers to discuss uncertainty with patients. Our synthesis yielded thirteen recommendations with three overarching aims: preparing for the discussion of uncertainty, informing patients about uncertainty and helping patients deal with uncertainty. Most recommendations lacked a solid empirical evidence base and were based on indirect evidence only.

Our synthesis reiterates what has been emphasized before: that there is no “one‐size‐fits‐all” approach to communicating about uncertainty and that providers’ use of the available strategies should depend on several characteristics of the situation.^5^ First, strategies varied in what *goal* they served. Some were specifically aimed at conveying information that involves uncertainty to patients and/or supporting patients in decision making. Others addressed how providers can help patients psychologically deal with the uncertainty and how they can respond when patients put forth their feelings of uncertainty. Such emotion‐ or relationship‐focused communication strategies may eventually help patients maintain hope and reduce their distress.^74^ We argue that often, these informational and emotional goals should be integrated rather than viewed in isolation or addressed consecutively. For example, after providers have explained the uncertainties regarding a specific clinical situation, it is crucial they support patients in dealing with them instead of abandoning them to make sense of and deal with uncertainty by themselves. For patients, being told that a diagnosis or test result is uncertain may be much more acceptable and/or bearable if providers provide strategies to deal with the uncertainty or a clear follow‐up plan. The communication of uncertainty is subservient to other goals: it is not an end in itself, and clinicians need to think about what the purpose is for any given patient and situation.

The second dimension on which recommendations varied is the *issue* to which they pertained. Whereas some applied to uncertainty in any clinical situation (eg, the advice to openly discuss the amount of available evidence), others were presented as specifically applicable to prognostic, diagnostic or therapeutic uncertainty. For example, the recommendations to prepare patients by highlighting future uncertainty and identifying suitable coping strategies for patients pertained specifically to situations involving genetic and diagnostic testing. This is not surprising, as clinicians discussing the use of genetic or diagnostic testing with patients are dealing with salient future uncertainty. Some recommendations may therefore be particularly suitable to specific clinical situations or types of health‐care providers. However, we observed few systematic patterns regarding the specific professions to which recommendations pertained, and even these more specific strategies may in practice be generalized across different situations. For example, clinicians involving patients in shared decision making may also want to prepare them beforehand to discuss uncertainty, to reduce any negative effects of uncertainty on patients.^23^

Third, optimal communication about uncertainty may depend on the *clinical scenario*. Particularly, life‐threatening and/or palliative situations may require specific strategies, such as providing a sense of control to counterbalance the uncertainty.^57^ A striking number of publications included in our review came from the fields of oncology and clinical genetics (see Table 2). Possibly, this reflects a heightened awareness of the many complex uncertainties inherent to these settings. However, uncertainty is highly prevalent in almost any clinical setting and many of the provided recommendations may therefore be applicable more widely.

The aforementioned goals, issues and clinical scenarios should always be taken into account when considering how to discuss uncertainty with patients. Strategies that work well in one situation may be less beneficial or even harmful in others. For example, explicitly outlining uncertainty may facilitate patient participation in treatment decision making (information‐focused goal). Yet, this increased awareness of uncertainty may provoke anxiety and reduce trust among vulnerable patients (emotion/relationship‐focused goal). Moreover, if providers openly acknowledge uncertainty about the future (eg, life expectancy, risk of relapse), this may be understandable and acceptable to patients, whereas conveying more complex uncertainty regarding the limits to one's present knowledge (eg, the accuracy of a diagnosis, the meaning of a test result) could require a more sensitive approach.^75^ Strategies may moreover interact, and it could be the combination and flexibility in using these various recommendations that yield the best results.

Clinicians additionally need to take individual differences between patients into account. For example, patients with lower health literacy may be less able to grasp complex uncertainties compared with others.^18^, ^76^ Additionally, highly emotional or anxious patients may benefit more than others if health‐care providers provide a sense of control to counterbalance uncertainty. Although we repeatedly encountered the advice to individualize communication, there were few practical suggestions available on how to do so. Future research should identify and substantiate optimal strategies for tailoring communication about uncertainty to individual patients. Additionally, moral discussion should address what amount of communication or non‐communication of uncertainty is appropriate. Such discussion may help providers reduce the tension between the moral imperative to convey uncertainty and the harmful effects it may have in some situations and/or individuals. This tension was visible in several seemingly contrasting recommendations we identified. For example, health‐care providers were on one hand advised to be open and explicit about the level of evidence and uncertainty, whereas on the other hand they were recommended to adjust the extent to which they convey uncertainty to individual patients, sometimes using more implicit rather than explicit wording (eg, “it is possible that…” instead of “I don't know”). This discrepancy may reflect the conflict between the normative goals of maximizing patient autonomy, that is informing about the limits of knowledge, vs enhancing patient well‐being, that is less explicit emphasis on potentially threatening uncertainties.^5^, ^77^, ^78^

Additionally, health‐care providers may need to tailor their communication strategies to their *own* feelings and behaviours in response to uncertainty, which may vary widely.^79^ Hence, certain strategies may work better for some than for others. For example, providers with lower tolerance for uncertainty may find particular benefit in explaining to patients the causes of uncertainty, emphasizing what *is* certain and/or making clear follow‐up plans. Alternatively, providers may need to be supported in tolerating uncertainty, which could eventually help them improve their clinical care.

Strengths of this review are, first, that we included both empirical and non‐empirical literature from a wide range of settings in our synthesis, and, second, our systematic search, selection and extraction, which enhances reliable and generalizable results. Third, by presenting the evidence base for all recommendations, readers can assess the underlying evidence for the available recommendations. Limitations are, first, that we may have excluded literature providing practical recommendations in the main manuscript only, and not in the title or abstract. Second, our synthesis may involve some degree of subjectivity. However, we attained maximal objectivity by continuously discussing our preliminary results within the full research group. Third, although we deliberately ignored the specific risk communication literature, recommendations from that area may be compared with the ones presented here to examine the extent of overlap and/or conflict.

The strategies for communication about uncertainty identified in this review align with and build on previous work,^47^ but more systematic empirical research is needed to substantiate them. Such empirical research should take into account that uncertainty communication can have various goals, and pertain to different issues and clinical settings. Additionally, research should clarify how health‐care providers can tailor their communication of uncertainty to individual patients and different situations. Eventually, this should lead to more specific practical advice for providers to flexibly adapt their communication style, thereby successfully navigating the tension between optimally informing about uncertainty and minimally harming patients by discussing uncertainty. Until then, health‐care providers may use our list of communication strategies as a useful starting point and toolbox to optimize their communication about uncertainty with patients.

## CONFLICT OF INTEREST

None.

## AUTHORS' CONTRIBUTIONS

Niki Medendorp: conceptualized the study, designed methodology, involved in formal analysis, investigated the data, curated the data, wrote the original draft, wrote, reviewed and edited the manuscript, visualized the data and administered the project. Cora Aalfs: conceptualized the study, and wrote, reviewed and edited the manuscript. Anne Stiggelbout: conceptualized the study, designed methodology, investigated the data, wrote, reviewed and edited the manuscript, and supervised the data. Paul Han: conceptualized the study, and wrote, reviewed and edited the manuscript. Ellen Smets: conceptualized the study, designed methodology, wrote, reviewed and edited the manuscript, supervised the data and acquired funding. Marij Hillen: conceptualized the study, designed methodology, involved in formal analysis, investigated the data, wrote the original draft, wrote, reviewed and edited the manuscript, visualized the data, supervised the data, administered the project and acquired funding.

## Data Availability

Data (literature search results and decisions on inclusion and exclusion) are available on request from the authors.
